# Supplementary material for: Determination of Selenium in Selenium—Enriched Products by Specific Ratiometric Fluorescence
Source: Sensors (Basel). 2023 Nov 15;23(22):9187. doi: 10.3390/s23229187 (PMC10674224; doi:10.3390/s23229187)
Supplement: Supplementary file 1 [file sensors-23-09187-s001.zip › sensors-2672513-supplementary.pdf]

Supporting information for

Determination of selenium in selenium - enriched products by specific ratiometric fluorescence

Munire Aimaitiniyazi [1], Turghun Muhammad [1, *], Ayzukram Yasen [2], Sainawaer Abula [3], Almire Dolkun [1] and Zulhumar Tursun [1]

[1] *State Key Laboratory of Chemistry and Utilization of Carbon-Based Energy Resources, College of Chemistry, Xinjiang University, Urumqi 830017, P. R. China.*
[2] *Key Lab of Natural Product Chemistry and Application, School of Chemistry and Chemical Engineering, Yili Normal University, Yining 835000, China.*
[3] *School of Safety Science and Engineering, Xinjiang Engineering Institute, Urumqi 830023, P. R. China.*
* Corresponding author: Turghun Muhammad, e-mail: turghunm@xju.edu.cn; Tel.: +8613669929903

Figure S1

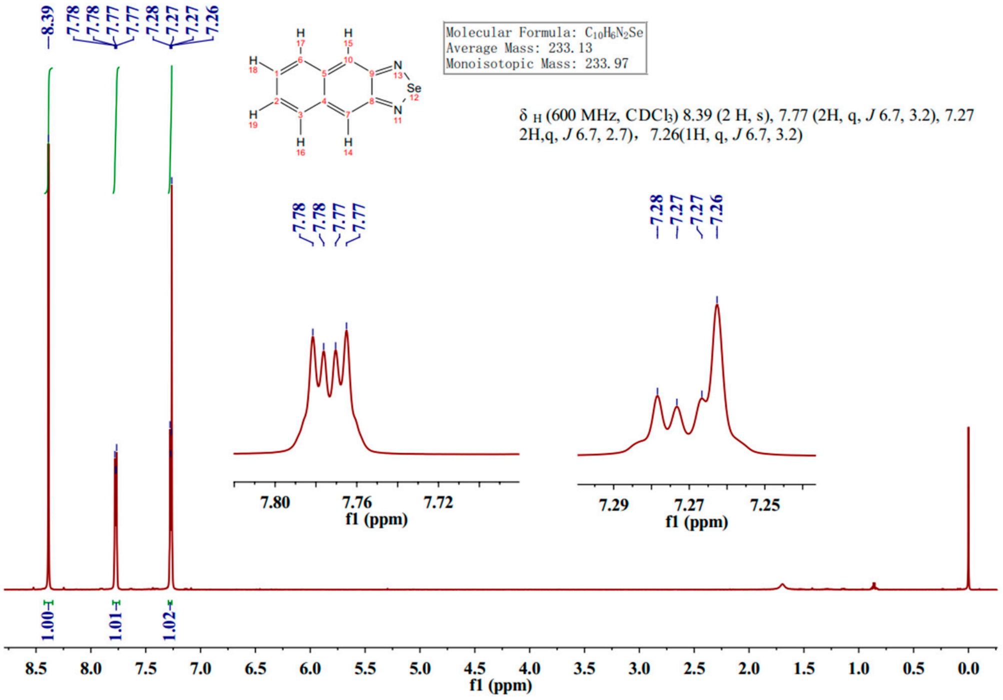

Figure S1. 1H-NMR spectrum of Se-DAN in $CDCl_3$.

Table S1

Table S1. Selenium content in shampoo (n=5)

Shampoo (g)	Se Content (mg. mL^{-1})		RSD (%)	
	FL	rFL	FL	rFL
0.50	6.17	8.19		
0.50	6.46	8.4		
0.50	6.97	7.91	6.63	3.02
0.50	5.84	8.57		
0.50	6.22	8.36		
**Average content**	6.63	8.29		

FL: Fluorescence method;

rFL: ratiometric fluorescence method;

Table S2

Table S2. Repeatability of results for selenium in milk samples (n=3)

Milk (mL)	FL (μg/mL)				rFL (μg/mL)			
	I	II	III	RSD (%)	I	II	III	RSD (%)
2.00	0.160	0.139	0.178		0.193	0.177	0.191	
2.00	0.163	0.134	0.144	11.9	0.203	0.183	0.189	5.18
2.00	0.179	0.134	0.139		0.198	0.175	0.179	
**Average**	0.167	0.136	0.154		0.198	0.178	0.186	

I, II and III represent the selenium content in milk measured at three different times.
